# Achieving a robust mentoring and research capacity program in a LMIC – the BRAINS faculty development model

**DOI:** 10.1186/s12909-023-04488-7

**Published:** 2023-07-20

**Authors:** Folasade Tolulope Ogunsola, Adekemi Sekoni, Alani Sulaimon Akanmu, Wasiu Lanre Adeyemo, Akinniyi Osuntoki, Bibiane Manga-Atangana, Bosede Bukola Afolabi, Njideka Ulunma Okubadejo, Madonna Emmanuel, Sikeade Olawumi Caleb-Adepoju, Olalekan Folarin, Prosper Okonkwo, Robert L Murphy, Phyllis Kanki

**Affiliations:** 1 Department of Medical Microbiology and Parasitology, University of Lagos, Lagos, Nigeria; 2 Department of Community Health and Primary Care, University of Lagos, Lagos, Nigeria; 3 Department of Haematology and Blood Transfusion, University of Lagos, Lagos, Nigeria; 4 Department of Oral and Maxillofacial Surgery, University of Lagos, Lagos, Nigeria; 5 Department of Biochemistry, University of Lagos, Lagos, Nigeria; 6 Adult Support Hub SIFA Fireside Birmingham United Kingdom, Birmingham, UK; 7 Department of Obstetrics and Gynaecology, University of Lagos, Lagos, Nigeria; 8 Department of Medicine, University of Lagos, Lagos, Nigeria; 9 BRAINS Initiative, College of Medicine, University of Lagos, Lagos, Nigeria; 10 Central Administration, APIN Public Health Initiatives, Abuja, Nigeria; 11 Havey Institute for Global Health, Feinberg School of Medicine, Northwestern University, Chicago, IL USA; 12 Department of Immunology and Infectious Diseases, Harvard TH Chan School of Public Health, Boston, MA USA

**Keywords:** Research, Training, Grants, Faculty, Mentoring

## Abstract

**Background:**

A research and training program (RTP) was carried out to build the capacity of faculty and improve the culture of research in the College of Medicine, University of Lagos (CMUL), Nigeria.

**Methods:**

Realist-guided mixed methods evaluation of the BRAINS project was carried out using secondary data generated during the 5-years (2015 – 2020) of project implementation. Capacity building workshops and mentored research activities targeted at faculty in the CMUL were conducted. Overall, 1,418 participants attended the workshops in batches. Among the participants, forty-five faculty received grants and were mentored by senior professionals (local & international) to conduct research. Data were extracted from all project-related documents including coursework biodata, workshop evaluation forms, quarterly project reports, and end- of-project reports, submitted by the mentees, minutes of meetings, and the proposal submitted for funding. It was in the form of continuous variables and prose (sentences & stories). Quantitative data were analysed with IBM SPSS statistics version 20. Mean knowledge score and mean difference was calculated, paired t-test was carried out using *p* < 0.05 to determine statistical significance. The prose was thematically analysed to generate themes and narratives. Both were subsequently combined for interpretation and used to refine the initial programme theory into an evidence-informed theory.

**Results:**

Twelve courses were deployed, and 1,418 participants (47.8% males and 52.2% females) from medical, nursing, and allied medical departments were trained. Eighty participants were trained in Responsible Conduct of Research and eighty-one on Manuscript Writing over three years. A comparison of the pre/post-test knowledge scores showed a positive mean difference. Thematic analysis of workshop data produced three thematic domains representing effectiveness and gains namely: cognitive, reward, and behavioural. 45 trainees were awarded grants and mentored, and analysis of mentee’s data generated 4 themes: Achieving a robust mentoring program; Benefits of the mentoring program; Resilience in research; Improving the mentoring program.

**Conclusion:**

By contributing to the body of knowledge available on RTPs, this evaluation identified key components that contributed to the success of the project and developed a model for achieving a robust training and mentoring program which can be replicated in other LMICs.

## Introduction

Several countries in the global south lack the capacity in areas like genomics and biomedical engineering to conduct the research necessary for developing innovative and locally relevant strategies to manage prevailing health-related problems. Building research capacity and improving the culture of R&D requires having access to training and development opportunities in addition to professionals who will support development and changes in professional practice [1, 2]. To fill this gap, the National Institutes of Health (NIH) Fogarty International Center (FIC) supports global health research and scientists and builds partnerships among research institutions and investigators in the United States and abroad including LMICs [3, 4].

Building Research and Innovation in Nigeria's Science (BRAINS) is a 5-year (2015–2020) program built on the achievements of the Medical Education Partnership Initiative Nigeria (MEPIN) funded by the Fogarty International Centre at the National Institutes of Health, Bethesda, USA. BRAINS is domiciled at the College of Medicine, University of Lagos (CMUL), in partnership with Northwestern University, Harvard University, and APIN Public Health Initiatives. Pre-COVID-19 pandemic, the BRAINS project provided training in the following areas of research; Human Immunodeficiency Virus (HIV) and infectious diseases, neuroscience, bioinformatics and genomics, community medicine, and biomedical engineering for junior faculty. The intention was to promote research and innovation, and in the spirit of inclusion and equality create avenues and platforms for academics to have access to and engage in cutting-edge research thereby raising local standards and grooming local academics for the competitive global stage [5, 6].

Innovation in healthcare leads to improvement in the quality of care and desired patient outcomes at the individual level. Harnessing of science in healthcare through research has led to the development of technology such as artificial intelligence and precision medicine resulting in improved healthcare delivery [7, 8]. Furthermore, multilevel, and trans-disciplinary approaches to research have been suggested as a suitable and more practical means of obtaining scientific findings that can be easily transformed into improved health outcomes. It is believed that this format will produce shared conceptual frameworks that can facilitate the implementation of scientific advances into practice [9].

The public policy-making process in the twenty-first century is expected to be evidence-based. However, the process is most often influenced by the agenda of politicians, issues of authority, power, and social, economic, and environmental risk considerations [10]. BRAINS project evaluation will contribute to the body of knowledge available on research training programs (RTP), this realist guided evaluation will also identify key components of RTPs that have been successful across different settings and ultimately contribute to the development of optimal and standard methods to evaluate global health RTPs. This evaluation aims to examine the input indicators, process indicators, output indicators, and outcomes of the projects to determine the project impact on the project beneficiaries.

## Methods

The executing institution is the College of Medicine, University of Lagos (CMUL), Nigeria. CMUL is a training center for healthcare students and professionals made up of three faculties. The faculty of Basic Medical Sciences with eight departments; The faculty of Clinical Sciences with thirteen departments; and The faculty of Dental Sciences with five departments.

This was a mixed methods study of BRAINS workshops and grant awards. All the lecturers in the CMUL below the rank of Asst Professor were eligible to participate in the program. The program office sent an email to all academic staff in CMUL advertising the workshops and mentorship research. The advertisement also included a registration link. Interested candidates who registered were requested to fill a biodata form and write a statement of purpose. For candidates who meet the eligibility criteria of junior faculty (rank of senior lecturer and below), the statement of purpose was screened and graded to assess suitability. Overall, 1,418 participants attended the workshops in batches. Year 1 to 4 workshops were in person while year 5 workshops took place during the COVID pandemic and were therefore virtual via Zoom. The sessions had slide presentations; small group discussions and presentations; lab practicals and plenary sessions depending on the course content. Pre-test questions were administered on day one of the workshops before the training commences while the post-tests were done on the last day of the workshop after completion of all the training content. The pre/post data included in the tables are for trainees that completed and submitted the pre and post-test for RCR and MW.

The workshop participants were encouraged to apply for the BRAINS mentored research grant which was open to all faculty in the CMUL. Among the workshop participants, forty-five faculty received grants and were mentored by senior professionals (local & international) to conduct research. The Responsible Conduct of Research and Grant Writing (RCR) course was used to evaluate objective 1a of the BRAINS program—Train faculty to ensure they are equipped to conduct research & develop projects; while the Manuscript Writing Course (MW) was used to evaluate objective 1b—Train faculty to produce strong manuscripts to present their findings. The mentees participated in some of the training workshops based on identified needs. Secondary data was collected from the project office, this consisted of information amassed during the 5 years of the project (routine data collected from trainees during coursework and workshop evaluation data, quarterly project report, and end-of-project report submitted by the mentees). The data were extracted using a Microsoft Word document in electronic format. All the BRAINS project documents are pass worded and kept secure. The data was in the form of continuous variables (quantitative) and prose (sentences).

Data entry was captured on Microsoft Excel, quantitative data was analysed with IBM SPSS statistics version 20. The general objective of the quantitative assessment was to evaluate the effectiveness of the training workshops using two courses that specifically addressed objective 1 of the BRAINS project. The data included in the analysis is for participants that completed and submitted the pre and post-test assessment. Mean knowledge score and mean difference was calculated, paired t-test was carried out using *p* < 0.05 to determine statistical significance.

The prose component of the data was analysed thematically using the Braun and Clarke technique [11]. Data analysis was carried out by 2 of the authors with initial independent reading and line-by-line open coding of the sentences followed by a meeting to review and agree on the codes. Using constant comparison, memos, and diagrammatic representations of the data, descriptive categories were developed, and subsequently condensed into themes based on emerging patterns. The qualitative and quantitative findings were subsequently combined for interpretation. The prose component was analysed to obtain facts regarding the context, enablers, and deterrents to the successful implementation of the project. It is considered an important step in the realist-inspired evaluation used for the BRAINS project.

This realist-inspired evaluation is tailored after the Pawson and Tilley approach [12], which emphasis identifying what works for whom in what circumstances (context + mechanism) to achieve outcomes. The evaluation commenced with the development of an initial program theory using the BRAINS proposal, planning, and execution documents to identify and define the process through which the program intended to improve the culture of research in CMUL. This initial theory uses a logic model i.e. a diagrammatic illustration of how coursework, grant funding, the practice of research, and pairing of junior faculty with experienced senior researchers will improve research culture and provide opportunities for locally relevant research (representation of the ideal concept of BRAINS). The essence of this is to explain the core elements of the programme while highlighting the logic of how the programme is intended to work. The next stage was to establish the progress of the programme towards set goals using secondary data supplied by the mentees and trainees that provided information on views and perspectives. This information was used to refine the initial programme theory into an evidence-informed theory by exploring the mechanisms by which mentoring as part of the BRAINS mentoring programme could impact research culture. The themes and narratives were incorporated to provide deep insights into the context within which the mechanism operated to generate predicted and unpredicted outcomes, to identify what worked or didn’t work as well as unravel the multilayer context responsible (Fig. 5).

## Results

**Fig. 1:**
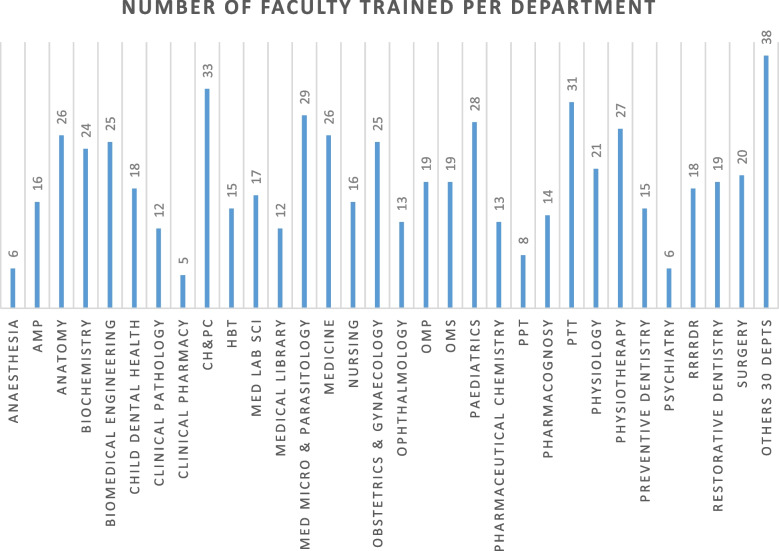
Distribution of trainees in CMUL by department. Pharmacology, Therapeutics & Toxicology (PTT); Pharmaceutics and Pharmaceutical Technology (PPT); Oral and Maxillofacial Surgery (OMS); Oral and Maxillofacial Pathology (OMP); Medical Microbiology & Parasitology (Med Micro & Parasitology); Haematology & Blood Transfusion (HBT); Medical Laboratory Science (Med Lab Sci); Community Health and Primary Care (CH&PC); Anatomic and Molecular Pathology (AMP)

Course work centred on twelve courses designed to build research capacity of junior faculty, 1,418 participants (47.8% males and 52.2% females) from medical, nursing and allied medical departments were trained (Table 1 and Fig. 1).

**Table 1:** Distribution of participants and courses attended

S/N	Course Title	Year 1		Year 2		Year 3		Year 4		Year 5		
		**Number of faculty**										
		**M**	**F**	**M**	**F**	**M**	**F**	**M**	**F**	**M**	**F**	**T**
1	Responsible Conduct of Research and Grant Writing (RCR)	33	20	10	16	10	11	21	25	11	14	171
2	Genomics and Bioinformatics Workshop	39	24	21	17	17	15	19	21	00	00	173
3	e-Learning	00	00	27	14	16	14	7	19	00	00	97
4	Manuscript Writing (MW)	00	00	24	15	22	22	13	17	7	20	140
5	Case-Based Method of Teaching	00	00	19	15	26	21	13	16	12	10	132
6	Public Health Research in Infectious Diseases	00	00	10	18	15	13	10	24	23	32	145
7	Research Design and Methodology	00	00	00	00	13	13	14	18	13	14	85
8	Bioinformatics	00	00	00	00	19	23	12	18	17	12	101
9	Mentoring	00	00	00	00	24	19	14	21	7	20	105
10	PowerPoint Presentation Skills Workshop	00	00	00	00	00	00	36	42	28	42	148
11	Data Analysis	00	00	00	00	00	00	16	25	17	17	75
12	Innovation	00	00	00	00	00	00	23	23	00	00	46

### Quantitative

Eighty participants were trained on RCR and eighty-one on MW over three years. Comparison of the pre and post-test knowledge scores showed a positive mean difference which was statistically significant for year 3 (*p* = 0.006) and year 4 (*p* = 0.001) RCR and year 5 MW (*p* = 0.001). The mean knowledge score for MW in year 3 showed a slight decline from 50.00 to 49.71 in the post test result. This decline was not statistically significant Tables 2 and 3.

**Table 2:** Responsible Conduct of Research (RCR)

Variable	Year 3	Year 4	Year 5
Number of Departments involved	16	22	15
Number of Participants with pre & post-test results	17	41	22
Mean knowledge score pre-test and post-test RCR	6.82 ± 1.185 8.0 ± 1.275	5.11 ± 2.303 6.33 ± 2.014	6.44 ± 1.199 7.11 ± 1.491
Year 3 Mean diff 1.176 ± 1.551 (CI 0.379 – 1.974) *p* = 0.006 Year 4 Mean diff 1.222 ± 1.675 (CI 0.655 – 1.789) *p* = 0.001 Year 5 Mean diff 0.667 ± 1.495 (CI—0.077 – 1.410) *p* = 0.076			
	%	%	%
Cadre			
Junior (Graduate Assistant, Assistant lecturer, Lecturer 2)	36	61	67
Senior (Lecturer 1 & Senior Lecturer)	63	39	33
Attendance at RCR course for the First Time	47	71	52
Gender			
Female	53	59	52
Young faculty			
Age ≤ 35	32	29	19
Statements that trainees strongly agreed with			
Attending the course is useful	95	81	86
Equipped to Conduct Research in-line with Best Practices	85	71	82
Understand how to Handle Conflict of Interest	75	56	77
Confident to Write & Apply for Grants	35	32	46

**Table 3:** Manuscript writing (MW)

Variable	Year 3	Year 4	Year 5
No of Departments involved	16	18	13
No of Participants with pre & post-test results	30	26	25
Mean knowledge scores pre and post-test	50.00 ± 2.774 49.71 ± 2.730	45.92 ± 7.533 47.92 ± 5.090	46.93 ± 4.649 51.00 ± 3.211
Year 3 Mean diff -0.286 ± 3.474 (CI -2.291 – 1.720) *p* = 0.763 Year 4 Mean diff 2.000 ± 6.083 (CI -1.676 – 5.676) *p* = 0.259 Year 5 Mean diff 4.071 ± 3.198 (CI—2.225 – 5.918) *p* = 0.001			
	%	%	%
Cadre			
Junior (Graduate Assistant, Assistant lecturer, Lecturer 2)	37	50	64
Senior (Lecturer 1 & Senior Lecturer)	63	50	36
Attendance at RCR course			
First Time	33	46	56
Gender			
Female	57	54	72
Young faculty			
Age ≤ 35	20	19	24

### Qualitative

Analysis of the RCR and MW workshop evaluation forms yielded three themes representing effectiveness and gains.

#### Theme 1—cognitive domain (68.8% of comments)

Trainees’ perception of empowerment revolves around knowledge to improve manuscript writing and a greater understanding of abstract optimization and referencing skills.

*I was able to acquire more elaborate understanding of manuscript writing especially on how I can optimize my abstract to increase visibility. In addition, the session on formatting references is very helpful. (Participant year 4)*

*I have significantly learnt how to write my manuscript and submit it for publication, I learnt about article optimization for the first time, also CONSORT Statement & RCTs. (Participant year 5)*

#### Theme 2—reward domain (21.1% of comments)

The trainees valued the contribution of the training to their promotion readiness self-assessment skill and ability to navigate promotion/career development hurdles.

*The session on the scoring system by the university has empowered me to get ready for my promotion, I learnt how to assess my promotion and the requirements involved; I acquired a new skill. (Participant year 3)*

*It has been very educative and an eye opener towards publication, promotion, and the policy of University of Lagos, understanding the process of evaluation of publications in UNILAG, I learnt more about the basic needs for my career development. (Participant year 4)*

#### Theme 3—behavioural domain (9% of comments)

Responses from participants indicated that the program helped motivate them to advance their careers.

*It has motivated me to complete unfinished manuscripts and encouraged me to re-submit some that I had abandoned due to rejection. (Participant year 3)*

*This course will help me to publish my unpublished work, I believe my next manuscript writing will be less stressful including writing a draft for peer review and overcoming my fear. (Participant year 5)*

Forty-five trainees ranging from assistant lecturers to senior lecturers across five faculties were awarded grants and mentored to conduct quality research (Table 4). Qualitative analysis of the mentees’ report generated four themes: Achieving a robust mentoring program, Benefits of the mentoring program, Resilience in Research, Improving the mentoring program (Table 5).

**Table 4:** Socio-demographic characteristics of the Mentees

Variable (*n* = 45)	Frequency (%)
*Gender*	
Female	23 (51.1)
Male	22 (48.9)
*Faculty of Mentees*	
Basic medical sciences	09 (20.0)
Clinical sciences	23 (51.1)
Dental sciences	05 (11.1)
Engineering related	02 (4.5)
Pharmacy	06 (13.3)
*Cadre of Mentees at the time of Award*	
Asst. Lecturer	02 (4.5)
Lecturer 2	06 (13.3)
Lecturer 1	18 (40.0)
Senior Lecturer	19 (42.2)
*Mentees with Established Collaborations*	
Yes	40 (88.9)
No	05 (11.1)
*Mentees who had Training Outside the Country*	
Yes	12 (26.7)
No	33 (73.3)

**Table 5:** Themes and Subthemes Mentees Qualitative Data

Themes	Subthemes
A. Achieving a robust mentoring program	*i. The mentoring experience was second to none* *ii. Building capacity of mentees through coursework* *iii. Funding academic research in a challenging economy* *iv. Grant management support*
B. Benefits of the mentoring program	*i. Improved research capacity* *ii. Networking & collaboration* *iii. Provided opportunities for upgrade (grantsmanship, conferences, professional development)*
C. Resilience in Research	*i. Challenges encountered* *ii. Innovative strategies developed*
D. Improving the mentoring program	

## Theme A: achieving a robust mentoring program

### Subtheme A (i)

The mentoring experience was second to none:* M*entors were considered very supportive because they provided encouragement and emotional support which kept the mentees motivated in the presence of challenges described as trying times. This support inspired and improved resilience ensuring that project objectives were achieved.

*The mentor taught me how to be focused, resilient, and determined to achieve my overall professional development. (R25)*

The mentors were a great source of useful information, advice & guidance on a wide array of subjects including the overall conduct of the research as well as highly technical scientific inputs. The advice given provided clarity and was useful in navigating thorny areas in research implementation. Areas mentioned by the mentees include manuscript drafting; obtaining approval from government institutions, ministries & agencies; lab work, analysis & interpretation of results; methodology (alternatives to face-to-face data collection during COVID19 lockdown); procurement; negotiation with suppliers; data collection tools; access to/recruitment of patients. Mentoring helped the mentees focus on achievable objectives and adhere to deadlines, this was achieved through periodic meetings and regular project reviews.

*My mentor was available ad libitum to provide guidance and a good critique of the research protocol with a guaranteed feedback mechanism that ensured a wonderful academic culture and a sustainable relationship that would last into the foreseeable future. The experience gained during this symbiotic relationship has formed a strong basis on which a sterling academic framework of discipline, attention to detail, and advancement of analytical mind would flourish. (R39)*

### Subtheme A (ii)

Building capacity of mentees through coursework: Mentees mentioned 13 key areas of capacity building because of attending the BRAINS coursework namely – Grant writing, Bioinformatics, Genomics, RCR, Research methods, Manuscript Writing, Data analysis, Networking & Collaboration, Mentorship, Ethics, Powerpoint Presentation, Innovation, and Project management. The highest five in ranking were Grant writing 20 (44%); Data analysis 13 (29%); RCR 12 (27%); Research Methods 11 (24%) and Manuscript writing 11 (24%).

*The BRAINS training contributed to my ability to conduct research in the areas of project planning, resources management, drive for grants, teamwork and team management, expanded my reach in terms of manpower accessibility, and has assisted me to develop both intellectual and physical project management prowess. (R3)*

*From attendee to facilitator, I have subsequently facilitated three times in the BRAINS research methodology workshop, which is also a support for my professional development. (R33)*

### Subtheme A (iii)

Funding academic research in a challenging economy: The BRAINS project commenced in 2015 with yearly grant awards over a five year period. The amount released to the mentees was based on the budget (in naira) requested in the proposal they submitted. Devaluation of the naira has been progressive in the past 7 years, resulting in rising costs of goods and commodities. This situation was exacerbated by the COVID-19 pandemic and subsequent global lockdown. Overall all the mentees were affected by the economic downturn but the overall impact of currency devaluation (up to half) and poor access to forex by importers was felt more by mentees with laboratory-based research work. Some of the mentees ran into financial difficulty during implementation and had to evolve innovative strategies to overcome funding related challenges. Four strategies were mentioned, reducing the scale of work; using personal funds; deploying funds from other grants; and negotiating further discounts with suppliers.

*More than 100% devaluation occurred with the grant money which was denominated in naira. The local arm of the study which involved the extraction of genomic materials was eventually shelved (R45)*

### Subtheme A (iv)

Grant management support: The grant management office was a pillar of emotional support to the mentees, the staff provided invaluable support to the smooth conduct of research work during the COVID-9 disruption. They provided encouragement and regular follow up which motivated the mentees and helped build resilience ensuring that mentees completed their research project within the specified period. Regarding practical support, the team assisted in the form of advice and guidance whenever required in several key areas including budgeting, documentation, and monitoring of progress, support with collecting the grant fund, report writing, project timelines, navigating difficult technicalities, registering with PubMed and eras common, community access and mobilization. This interactive process built the capacity of mentees in project management skills. BRAINS project staff ensured that mentees were informed and supplied with reminders regarding other grant opportunities & applications. Access to such vital information was crucial in the harvest of grants witnessed by the mentees. Effective communication with regular feedback and prompt response to clarifications, when needed, ensured challenges were handled in an effective and efficient manner.

*The BRAINS research office provided timely information about what was expected of me as a mentee. They facilitated the smooth release of the fund approved for the study. They were passionate to get updates about the progress of the work. (R15)*

The grant management office also provided additional financial support outside of the grant for mentees to present their findings at an international conference; travel to collect a Centennial award; purchased the license for statistical software thereby paving way for a more robust data analysis and reporting of research findings.

## Theme B. Benefits of the mentoring program

### Subtheme B (i)

Improved research capacity: Conducting research as a principal investigator was an exciting experience for the mentees. This research independence provided a great learning opportunity, practicing old skills and acquiring new skills resulting in improved research capacity and research work of an international standard. Learning was continuous and cut across a range of subjects. Mentees believe that the BRAINS program greatly changed how they conducted research. For faculty working on genomics related research, the knowledge gained from the training was invaluable, the exposure and skills acquired from the bench work provided a foundation that can be applied to research on genomics of Infectious diseases like HIV/AIDS, TB, and other microbial diseases endemic to Nigeria. In addition, researchers experienced the unique challenges inherent in conducting research in their various fields of specialization.

Mentees acquired skills in the following areas: Grant/proposal writing, Data analysis, Manuscript writing, Budgeting, Project management, Networking & Collaboration, Ethics, Genomics and RCR. Ranking with regards to importance statistical analysis 38%; Genomics 36%; Project management 33%; Grant writing 27% and Manuscript writing 24%. Other areas of enhanced capacity include ability to come up with research questions, conceptualise, design and conduct scientific research; research management; communication; critical thinking; innovative data collection techniques (data collection in batches); medical device product development; laboratory skills in quantitative RTPCR and Western Blot.

*This mentored program has helped me to build skills and capacity that are useful in executing research while adapting to the use of resources available in a low-cost environment as well as collaborating with experts from other fields to bring out the best in my research. (R24)*

*If I am to put everything on a rating scale, I would say that the BRAINS has improved my ability to conduct research from 40% to 90%. I will attribute this success to the skills acquired in grant writing from the BRAINS workshop; and the fact that this BRAINS grant boosted my biosketch. (R9)*

### Subtheme B (ii)

Networking & Collaboration: Mentoring exposed the mentees to improved networking opportunities and active multidisciplinary collaboration locally and internationally. For some mentees it provided access to a wider network of diverse but relevant collaborators which facilitated access to experts in various fields, laboratory facilities, and information regarding conferences.

*Provided me the opportunity to collaborate successfully with experts in various fields, lead a team composed of professionals across different cadres, broadened my knowledge of biomaterials and increased my innovative capacity. The opportunity of joining an additional research team headed by one of my collaborators has resulted in co-authoring in a chapter in a biomaterials book, a manuscript and an application for two other grants. The BRAINS funding opportunity has resulted in a marked improvement in my profile and opened opportunities for future collaborations. (R22)*

### Subtheme B (iii)

**Fig. 2:**
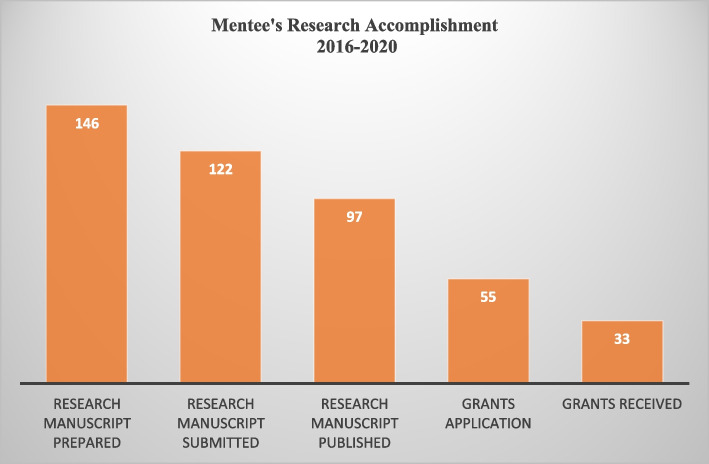
Manuscript and grant related accomplishment of BRAINS mentees

**Fig. 3:**
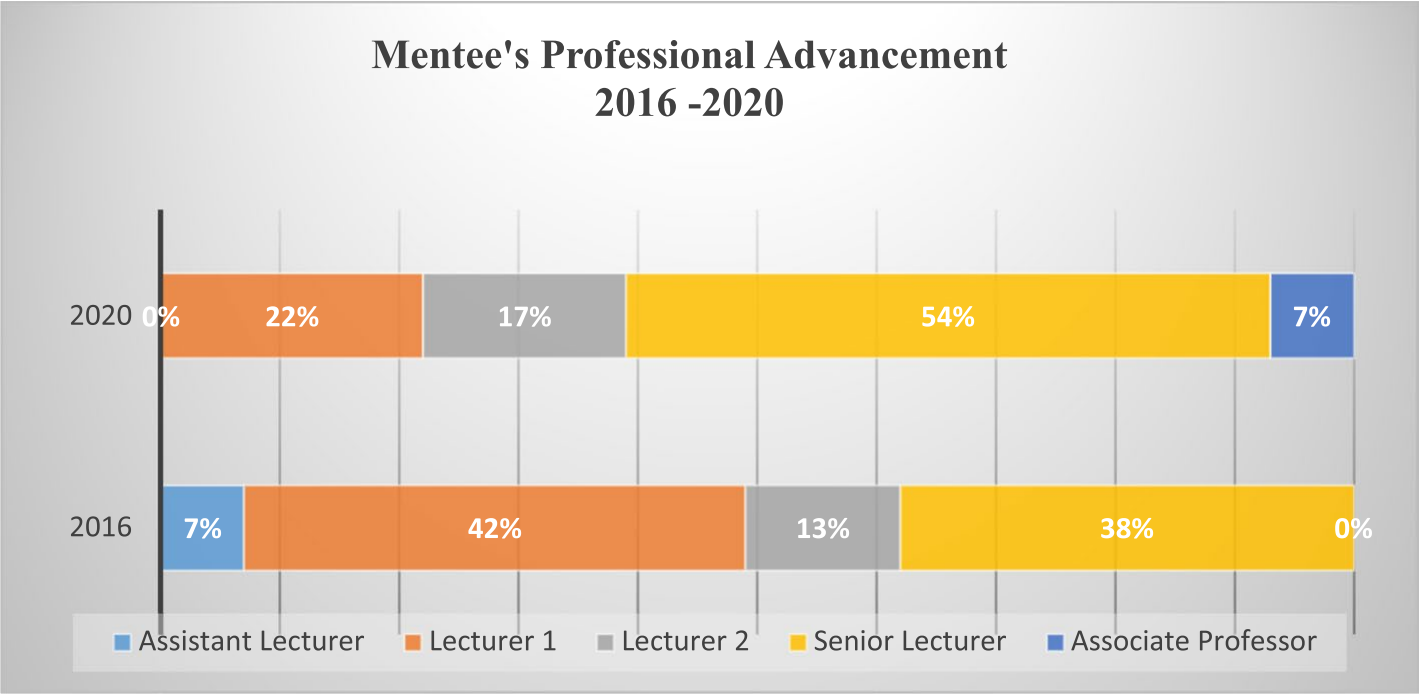
Professional advancement of BRAINS mentees

Provided opportunities for upgrade (grantsmanship, conferences, and professional development/promotions): Figs. 2 and 3.

Going through the BRAINS mentoring program built the personal confidence of the mentees resulting in an improved ability to write and execute translational and ethical research projects. The increased personal confidence made it possible for mentees to apply for international conferences and grants which were largely successful. Application of due diligence and prudence in managing scarce resources in the execution of such research projects contributed to excellent project management. Some mentees were also able to develop a research direction. Coupled with the capacity building that was a key feature of the program, at the end of the mentoring period, it was a natural phenomenon for some individuals to transition from mentees to mentors.

*The mentored program has ensured that I also am a mentor. I have learnt from my mentor and I am now mentoring others to get grants. One of my mentees received a grant this year. (R4)*

*The BRAINS Mentored research afforded me the opportunity to conceive a fundable research grant proposal in a more organized and methodical way. I was able to submit this proposal for a K43 award and I got the award. (R20)*

## Theme C. Resilience in research

### Subtheme C (i)

Challenges encountered: The research environment was challenging largely due to the unavailability of some equipment, reagents and materials locally. Therefore researchers had to contend with procurement, supplies and repair related difficulties. Coupled with irregular electricity supply, the environment was unconducive for twenty-first century research activities. Obtaining approvals from relevant government institutions and agencies for research activities was reported to be bedevilled with time consuming bureaucracy resulting in delays in patient recruitment, data collection, app development etc. leading to missed deadlines and delays in achieving research objectives. Additionally, the COVID19 pandemic related lockdown brought a unique set of challenges to research implementation (including laboratory work; data collection; patient recruitment, procurement & supplies) and excess workload. Also, most of the researchers are healthcare workers whose workload increased tremendously during this period.

### Subtheme C (ii)

Innovative strategies developed: Mentees dealt effectively with the challenges mentioned by proactively adopting several relevant innovative problem solving strategies for implementation as well as maximising local/international collaboration efforts. This involved extended multicentre data collection plans; using more research assistants; traveling abroad for the lab work; moving samples to more efficient centres; reducing the scale of work; assistance from colleagues and collaborators for recruitment, procurement, lab work and data collection. Researchers were of the opinion that character traits of patience and persistence came in very handy in handling the very difficult and stressful situations they had to endure.

*Colleagues in the (mentions name of the lab) helped me complete the laboratory processes while I had to return to Nigeria because of the pandemic (R36)*

*My mentor gave access to the pathology laboratory and also put me in contact with a junior faculty in pathology with whom I was able to perform the pathology aspects of the project (R41)*

## Theme D. Improving the mentoring program

The BRAINS mentored research program was highly rated by the mentees however, areas of improvement for future programs were mentioned. The recommendations revolved around mentors and collaborations; and secondly training and technical support. Mentees were of the opinion that having one local & one international mentor per mentee was very beneficial boosting collaborative efforts especially with other laboratories and highly skilled experts. In addition to the current bouquet of courses mentees requested for additional international training in molecular and genomic studies; qualitative methods and mixed methods study design; practical laboratory training. Technical support in dealing with the bureaucracy surrounding payment to foreign suppliers by the central bank of Nigeria and approval from other relevant institutions was also considered vital.

*Support in fast tracking the process of patent application & filing. Support in reducing the delays by Central Bank of Nigeria in making payments to foreign suppliers (R14)*

## Discussion

**Fig. 4:**
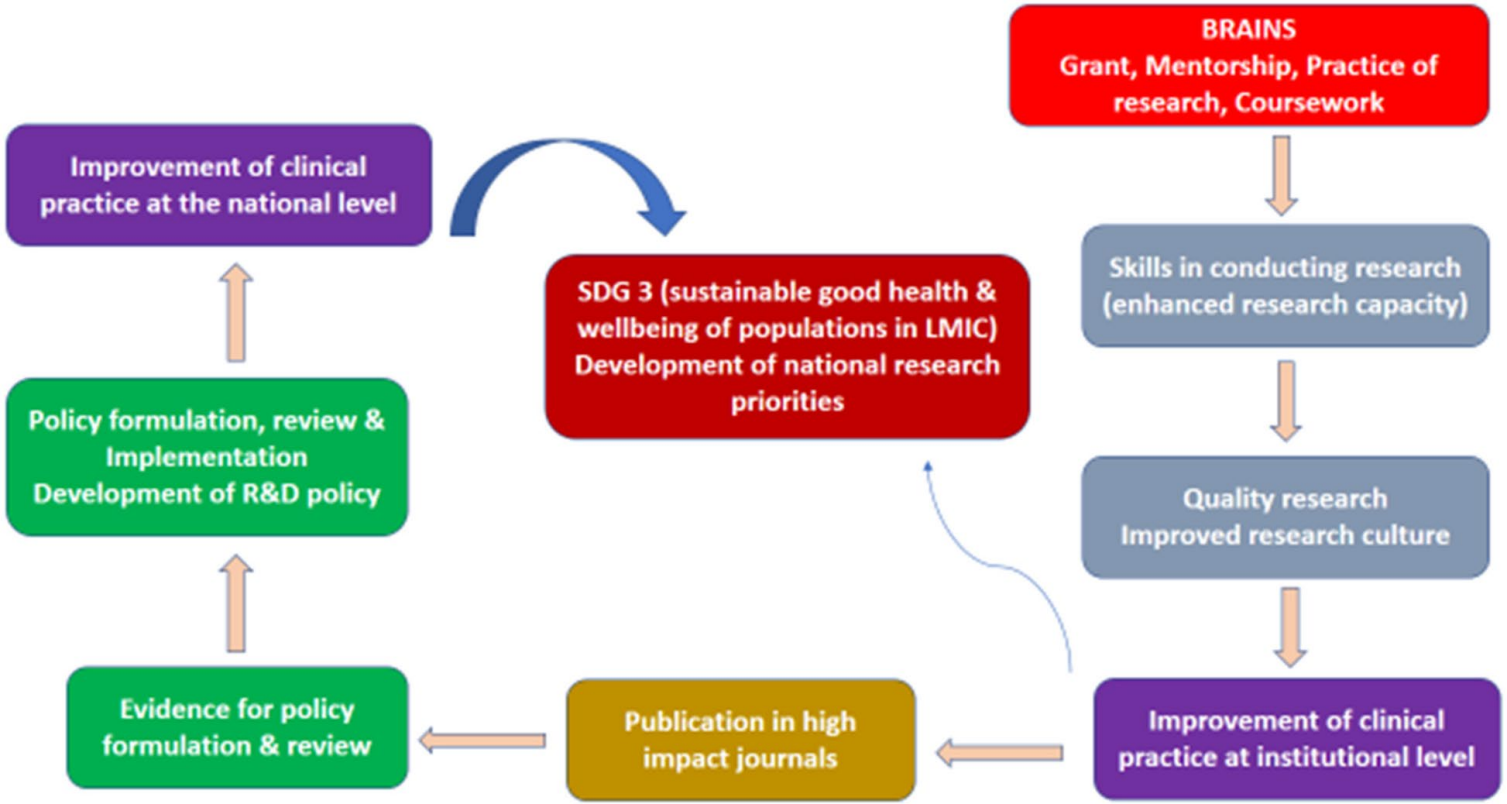
Projected Impact of Brains Mentored Programme

This realist-guided program evaluation integrated qualitative analysis with some quantitative measurements to assess the impact of a formal and collaborative research training and mentoring program (RTMP) on building the research capacity of faculty in a sub-Saharan African country. In keeping with expectations from the funders and reports from previous Fogarty training programs, our RTMP demonstrated a high return on investment and other downstream benefits [4, 13, 14]. The major success indicators for improvement in individual research training capacity include the number of manuscripts submitted to journals and subsequently published in high-impact journals; acquisition of skills in conducting quality research and enhanced research capacity of the health workforce which will ultimately lead to improved research culture in the University (Fig. 2). These performance metrics are interwoven and constitute a continuous sustainable cycle because the scholarly output will ensure access to vital information necessary for policy formulation/review and improvement of clinical practice at the institution and country-wide (Fig. 4).

Publishing research in peer review journals is an important step in sharing and disseminating credible knowledge and information. Publishing quality research shows that the grant funds have been judiciously applied and therefore an invitation for bigger grants to build on what has been achieved. At the same time, publications provide evidence that the research is ethical, and has been designed, carried out, analysed, and written up to a standard that is acceptable to the global scientific community [15–17]. Other downstream benefits from the RTMP include multidisciplinary research collaboration with international partners, grants from other funders, and career advancement of the mentees. Our finding is in consonance with reports of previous program evaluations from other LMICs reporting individual-level research training outcomes namely publications/dissemination of study results, conference presentations, and successful grant applications [18–22]. One of the pivots of this program was mentorship from local and international specialists. Each of the mentors contributed richly to the project and improved both the capacity of junior researchers and the research environment through the introduction of skills, expertise, use of facilities & laboratories, and diverse work cultures. The major strength of BRAINS is local leadership made up of a multidisciplinary team a proven crucial determinant of sustainability of the gains of RTMP and other funded schemes in LMIC [23–26].

## Conclusion

**Fig. 5:**
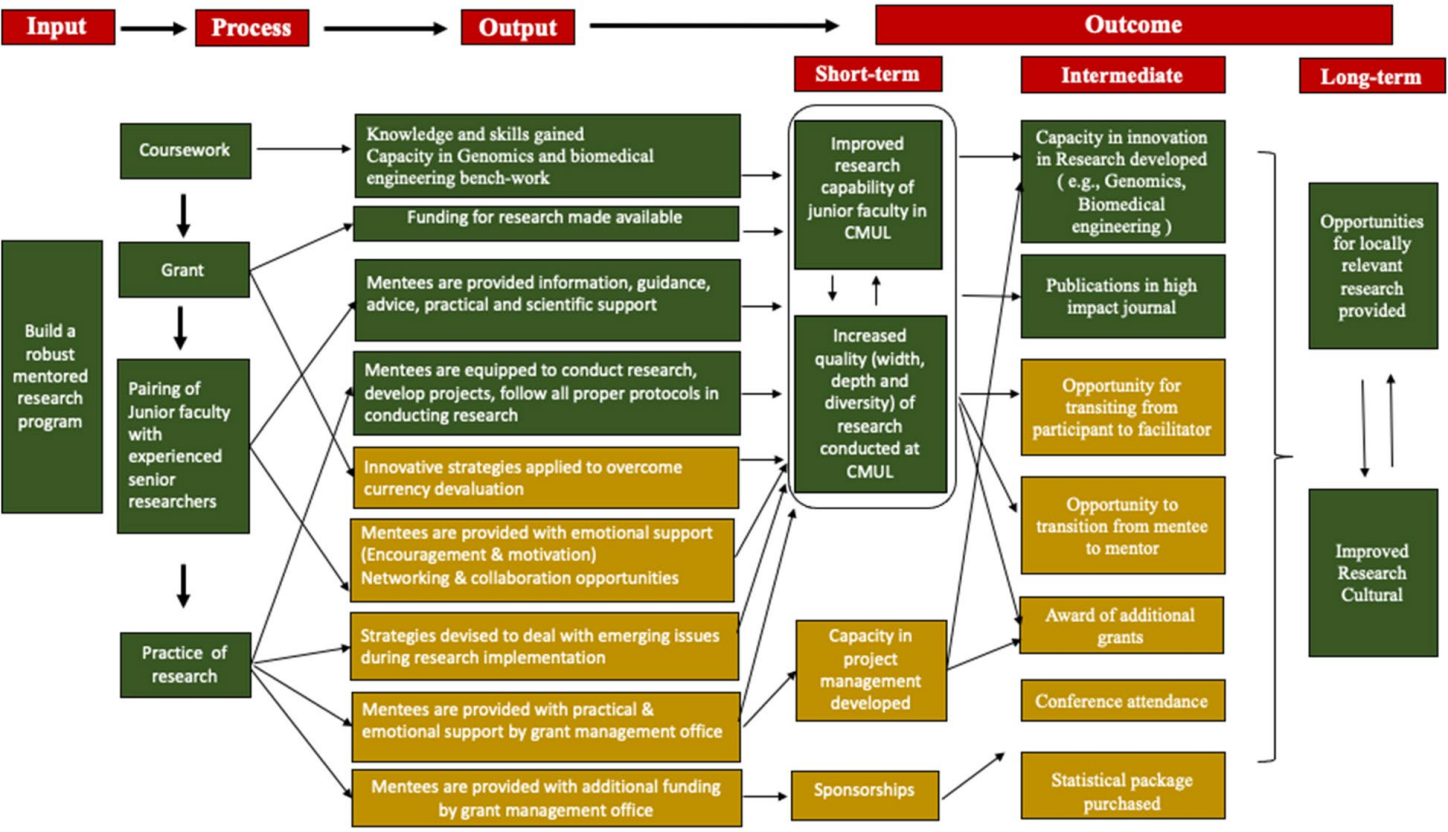
BRAINS Model for Achieving a Robust Mentored Research Program

The BRAINS project succeeded in bringing together a large number of experienced and skilled specialists locally and in the global north to mentor and build the research capacity of junior faculty in CMUL. This fostered relationships at individual and institutional (Universities) levels, each institution bringing a different set of skills and expertise, competencies, and diverse work cultures to enrich the project. The interaction between the actors gave birth to more funded proposals and grants, publications in high-impact journals, research activities, and the professional advancement of individuals (Fig. 5). The findings from this evaluation will strengthen and improve the scale-up of capacity-building projects to other similar institutions.

Recommendations: In as much as the aim of this mentored research program was to improve the research culture of individual faculty, it is important to design tools for measuring institutional research capacity. This will allow the project team captures the effectiveness of the project by determining the degree of change at specific timelines. The COVID-19 pandemic has shown that going forward research needs and capacity will have to evolve at a faster rate necessitating programs to continuously conduct surveys to identify perceived needs and gaps. This helps to ensure that the training remains relevant and at the same time, this is a great step to maintain and consolidate the research culture for sustainability at the institution; Knowledge sharing and dissemination through ongoing research methodology and proposal & manuscript writing activities at the institutional level for all PG students by the mentees will extend the gains beyond the institution for easy scale-up of improvement countrywide; It is equally important to build a sustainable enabling environment to retain highly motivated scholars and skilled researchers.

## Data Availability

The data that support the findings of this study are available from the authors and the Grants Office College of Medicine, University of Lagos, Nigeria. Request should be addressed to: Prof Folasade Tolulope Ogunsola (PI) Email: sade.ogunsola@gmail.com
